# Mitochondrial DNA copy number in affected and unaffected LHON mutation carriers

**DOI:** 10.1186/s13104-018-4025-y

**Published:** 2018-12-20

**Authors:** Angelica Bianco, Alessio Valletti, Giovanna Longo, Luigi Bisceglia, Julio Montoya, Sonia Emperador, Silvana Guerriero, Vittoria Petruzzella

**Affiliations:** 1 Dipartimento di Scienze Mediche di Base, Neuroscienze e Organi di Senso - Università degli Studi Aldo Moro, Piazza G. Cesare, 70124 Bari, Italy; 2 Ospedale Casa Sollievo della Sofferenza IRCCS, UOC Genetica Medica, San Giovanni Rotondo, Italy; 3 Departamento de Bioquímica y Biología Molecular y Celular, Universidad de Zaragoza-CIBER de Enfermedades Raras (CIBERER)-Instituto de Investigación Sanitaria de Aragón (IIS Aragón), 50013 Zaragoza, Spain

**Keywords:** Incomplete penetrance, Leber’s hereditary optic neuropathy, Mitochondrial genome, mtDNA copy number

## Abstract

**Objectives:**

Leber’s hereditary optic neuropathy (LHON) is a mitochondrial genetic disease characterized by a variable and reduced penetrance. Individuals carrying a primary LHON-causing mitochondrial DNA (mtDNA) mutation may either remain asymptomatic lifelong, as unaffected carriers, or develop sudden central visual loss that rapidly aggravates over some weeks. Over the years several genetic/environmental triggers able to modulate the risk of developing LHON have been proposed. We provided data supporting a possible correlation between LHON penetrance and the mtDNA copy number, a raw index of mitochondrial mass, whose increase could represent a compensatory response that cells implement to alleviate the pathogenic effect of the primary LHON-causing mtDNA mutations.

**Data description:**

We collected Italian and Spanish subjects harboring one of the three common LHON primary mutations, either in heteroplasmic or homoplasmic status. For each population we were able to discriminate between affected subjects presenting typical clinical tracts of LHON and LHON-causing mutation carriers showing no symptoms correlated with vision loss. Each subject has been characterized for the presence of a LHON primary mutation, for its status of homoplasmy or heteroplasmy, and for the mtDNA content per cell, expressed as relative mtDNA/nDNA ratio respect to controls. Additional clinical information is present for all the Italian subjects.

## Objective

Leber’s hereditary optic neuropathy (LHON) is typically characterized by a rapid bilateral central vision loss owing to focal degeneration of the retinal ganglion cell layer and optic nerve [1, 2]. The presence of primary mutations in mitochondrial DNA (mtDNA) is necessary, but not sufficient alone, to cause optic neuropathy, because disease penetrance can even vary within different families harboring the same mutation [3, 4]. Thus, the idea that other environmental and/or genetic factors might affect the penetrance and the risk of developing LHON is being reinforced over the last years [5, 6]. Nonetheless, when the etiology of a disease involves mitochondrial mutations it is mandatory to consider that the mtDNA is a multi-copy genome whose cell quantity varies depending on tissue type and pathophysiology factors. Furthermore, adjustment of the mtDNA content can represent a protective strategy cells perform to compensate whatever detrimental effect a mtDNA mutation is causing, whose efficacy is experimentally proven [7–9]. For instance, mitochondrial proliferation is commonly seen in post-mitotic tissues such as skeletal muscle in patients with mitochondrial disease [10]. The mtDNA copy number can be assessed in peripheral blood and is thought to reflect variations in mitochondrial energetic function and biogenesis occurring in other tissues otherwise unaccessible for diagnostic tests [11].

The purpose of the data collected was to provide support to a possible correlation between the mtDNA levels and LHON penetrance in a population harboring a primary LHON-causing mutation. As already reported in other studies [12–16], unaffected mutation carriers showed the highest amount of mtDNA, regardless of the heteroplasmic/homoplasmic status. Furthermore, we observed that the mtDNA copy number progressively shifted towards higher values from controls to carriers, with the affected showing an intermediate value. This could suggest that in both carriers and affected individuals there is an activation of the mitochondrial biogenesis, somehow hindered in affected subjects.

## Data description

We collected 124 subjects with a primary LHON-causing mutation (i.e., m.11778G > A or m.3460G > A), of which 51 Italians and 73 Spanish. Two different control groups were considered, specifically 90 unrelated Italian healthy subjects and 28 unrelated Spanish healthy subjects (Table 1—Data set 1–3) [17–19], the latter used only for the analysis of the homoplasmic Spanish population as this was analyzed in a different laboratory, even if following the same general procedures.

**Table 1:** Overview of data sets

Label	Name of data file/data set	File types (file extension)	Data repository and identifier (DOI or accession number)
Data set 1	Italian subjects with a LHON-causing mutation in homoplasmy [13, 17]	MS Excel file (.xlsx)	Figshare (10.6084/m9.figshare.7093559.v1)
Data set 2	Spanish subjects with a LHON-causing mutation in homoplasmy [15, 18]	MS Excel file (.xlsx)	Figshare (10.6084/m9.figshare.7093619.v1)
Data set 3	Italian and Spanish subjects with a LHON-causing mutation in heteroplasmy [14, 19]	MS Excel file (.xlsx)	Figshare (10.6084/m9.figshare.7093643.v1)
Data file 1	Methods [21]	MS Word file (.docx)	Figshare (10.6084/m9.figshare.7133840.v3)

On the basis of clinical features and genetic mitochondrial analysis, we identified 46 Italians subjects, belonging to 20 families, carrying a LHON-causing mutation in homoplasmy (37 m.11778G > A, distributed between 18 affected and 19 carriers, and 9 m.3460G > A, of which 5 affected and 4 carriers) (Table 1—Data set 1) [17] and 52 Spanish (27 m.11778G > A, distributed between 18 affected and 9 carriers, and 25 m.3460G > A, of which 6 affected and 19 carriers) (Table 1—Data set 2) [18].

We also identified 26 subjects (Spanish and Italians), belonging to 12 families, carrying a LHON-causing mutation in heteroplasmy, distributed as follows (Table 1—Data set 3) [19]: 9 subjects with the m.11778G > A mutation (1 affected and 8 carriers), and 17 subjects with the m.3460G > A mutation (4 affected and 13 carriers). The mutant allele frequency was variable, ranging from 30 to 95% and from 5 to 95% for m.11778G > A and m.3460G > A, respectively.

These subjects were already partially described in our previous papers [13–15].

Subjects were collected at three sites: Ophthalmology Clinic, Policlinico Bari Hospital, Italy; Hospital IRCCS ‘Casa Sollievo della Sofferenza’, Italy; and the University of Zaragoza, Spain. Prior written and informed consent was obtained from each subject according to Institutional Guidelines. Several examinations were performed: slit-lamp biomicroscopy, fundal and optic nerve head stereoscopy, fluorescein angiography, optical coherence tomography, and visual field testing. Total genomic DNA was extracted using the “Wizard^®^ Genomic DNA Purification Kit” (Promega) from peripheral blood of the patients and their relatives with suspicion of LHON and from healthy control subjects. The presence of LHON mutations (m.3460G > A, m.11778G > A and m.14484T > C) was detected by PCR-RFLP and, if present, confirmed by direct sequencing (ABI prism 310, Applied Biosystems). Quantification of mtDNA copy number was performed by qPCR using the relative method [20]. Mitochondrial and nuclear DNA quantities were measured amplifying genomic regions of ND1 and B2M genes, respectively. mtDNA/nDNA ratio was calculated for each sample and this value was then calibrated relative to the geometric mean of controls (i.e., relative mtDNA/nDNA ratio). For further details see Data file 1 (Table 1) [21].

Summing up the homoplasmic population data, in Italian subjects the mtDNA content (measured as relative mtDNA/nDNA ratio and expressed as geometric mean and confidence interval CI) was 100 (CI 93.38, 107.09) in controls, 133.72 (CI 112.34, 159.17) in affected and 173.97 (CI 140.53, 215.37) in carriers; in Spanish population we measured these mean values: controls 100 (CI 83.79, 119.35, affected 104.29 (CI 86.02, 126.44), and carriers 147.73 (CI 124.82, 174.84).

Heteroplasmic subjects showed these relative mtDNA/nDNA ratio mean values: controls 100 (CI 93.38, 107.09), affected 140.25 (CI 89.51, 219.75) and carriers 234.54 (CI 197.45, 278.61).

## Limitations

These data do not consider other known variables, such as mtDNA haplotypes and nuclear genetic background that represents likely triggers of LHON and are known to influence penetrance and age of onset.

## Abbreviations

LHON: Leber’s hereditary optic neuropathy
mtDNA: mitochondrial DNA
nDNA: nuclear DNA
CI: confidence interval
